# The Impact of Concussion, Sport, and Time in Season on Saliva Telomere Length in Healthy Athletes

**DOI:** 10.3389/fspor.2022.816607

**Published:** 2022-02-15

**Authors:** Matthew Machan, Jason B. Tabor, Meng Wang, Bonnie Sutter, J. Preston Wiley, Richelle Mychasiuk, Chantel T. Debert

**Affiliations:** ^1^Department of Clinical Neurosciences, University of Calgary, Calgary, AB, Canada; ^2^Faculty of Kinesiology, University of Calgary, Calgary, AB, Canada; ^3^University of Calgary Sport Medicine Centre, Faculty of Kinesiology, University of Calgary, Calgary, AB, Canada; ^4^Department of Neuroscience, Monash University, Melbourne, VIC, Australia

**Keywords:** sport-related concussion, athletes, fluid biomarkers, saliva, telomere length

## Abstract

To date, sport-related concussion diagnosis and management is primarily based on subjective clinical tests in the absence of validated biomarkers. A major obstacle to clinical validation and application is a lack of studies exploring potential biomarkers in non-injured populations. This cross-sectional study examined the associations between saliva telomere length (TL) and multiple confounding variables in a healthy university athlete population. One hundred eighty-three (108 male and 75 female) uninjured varsity athletes were recruited to the study and provided saliva samples at either pre- or mid-season, for TL analysis. Multiple linear regression was used to determine the associations between saliva TL and history of concussion, sport contact type, time in season (pre vs. mid-season collection), age, and sex. Results showed no significant associations between TL and history of concussion, age, or sport contact type. However, TL from samples collected mid-season were longer than those collected pre-season [β = 231.4, 95% CI (61.9, 401.0), *p* = 0.008], and males had longer TL than females [β = 284.8, 95% CI (111.5, 458.2), *p* = 0.001] when adjusting for all other variables in the model. These findings population suggest that multiple variables may influence TL. Future studies should consider these confounders when evaluating saliva TL as a plausible fluid biomarker for SRC.

## Introduction

Mild traumatic brain injury (mTBI) is a prevalent health concern, affecting almost 34 million people worldwide and an estimated 350,000 Canadians annually (Arciniegas et al., 2005; Brain, 2007; Prins et al., 2013; Gardner and Yaffe, 2015). The third leading cause of mTBI is sports and recreation (Canadian Institute for Health Information, 2006), with up to 15% of athletes reporting one or more sport-related concussions (SRC) in their season (Harmon et al., 2013). The international consensus statement on concussion in sport recommends assessment of acute SRC with the sport concussion assessment tool-5 (SCAT5) (McCrory et al., 2016). The SCAT5 is best employed within the first 48 h of injury for diagnostic purposes, significantly losing diagnostic ability beyond 3 days post-injury (however is still useful for tracking recovery using the embedded symptom scale) (Echemendia et al., 2017). In conjunction with the SCAT5, a multi-model approach is recommended as this may increase diagnostic and prognostic ability (McCrory et al., 2016). Other diagnostic modalities, such as imaging and fluid biomarkers, hold promise but are still in their infancy of diagnostic accuracy for SRC. Recent studies have shown metabolite profiling (Daley et al., 2016; Wanner et al., 2021), inflammatory cytokines (Nitta et al., 2019; Battista, 2020a,b), tau protein (Shahim et al., 2016), s100B protein (Kiechle et al., 2014), neuron specific enolase (NSE) (Shahim et al., 2014), AMPAR peptide (Dambinova et al., 2013), glial fibrillary acidic protein (GFAP) (McCrea et al., 2019), ubiquitin C-terminal hydrolase-L1 (UCH-L1) (Meier et al., 2020), prolactin levels (Schulte et al., 2013), plasma soluble prion protein (PrPC) (Pham et al., 2015) and cortisol (Ritchie et al., 2018) may be useful blood biomarkers to adjunct current tools for management of SRC. However, no definitive fluid biomarkers have been recommended for clinical application. To better understand the utility of fluid biomarkers for diagnosis and prognosis, exploration of the biomarker of interest requires extensive investigation in a non-injured population. Once an understanding of the influence of confounders and normative ranges are established, investigating variance of the biomarker of interest in an injured population can occur. We therefore sought to examine a fluid biomarker of interest, saliva telomere length (TL), in an uninjured varsity athlete population to explore the effects of multiple common confounders. Damage to the central nervous system (CNS) may be reflected in saliva TL (derived from buccal cell DNA), as brain damage and a compromised blood-brain barrier induces an increased immune response and consequently systemic oxidative stress to cells in the periphery (Friedrich et al., 2000; Hehar and Mychasiuk, 2016; Aaron Dadas, 2018; Demanelis et al., 2020).

Telomeres are repetitive non-coding DNA sequences on the ends of linear chromosomes that protect cells and DNA from erosion during cellular replication, or from damage that would trigger apoptosis. Telomeres also influence epigenetic patterning, DNA repair, and cellular growth (Eitan et al., 2014; Hehar and Mychasiuk, 2016; Hehar et al., 2017). Under normal conditions, each successive cell division causes a cell's telomere length (TL) to shorten ~30–200 base pairs; the shorter the TL, the closer the cell is to senescence (Starkweather et al., 2014). Decreases in TL are typically associated with aging, but TL may also be influenced and potentially expedited by lifestyle, environmental factors, biological factors, and an individual's injury history (Starkweather et al., 2014).

Injury to the CNS induces oxidative stress and inflammation and can result in neurodegeneration. Neurodegeneration may subsequently cause damage to TL as well as downregulate telomerase, the enzyme responsible for maintaining telomeres and facilitating repair (Smith et al., 2013; Eitan et al., 2014; Hehar and Mychasiuk, 2016; Wright et al., 2018). CNS injury occurring during SRC influences TL. Preliminary studies suggest that assessment of TL following concussion may aid diagnosis and prognosis, by providing insight into the pathophysiological changes that occur. A preclinical study by Mychasiuk et al., examined the association between brain damage and TL using a clinically-relevant rodent model of concussion. The authors found a single concussion significantly reduced TL in serum compared to sham injury (Hehar and Mychasiuk, 2016; Hehar et al., 2017). These findings translated into clinical research, where Symons et al. found that on average, Australian football players had shorter saliva TL than athletes participating in non-contact sports (Symons et al., 2020). Further, a recent systematic review found individuals that participated in aerobic exercise had longer telomeres than inactive controls – especially when the exercise was moderate to high intensity (Lin X. et al., 2019). The mode of physical activity or ratio of aerobic to anaerobic exercise in each sport is likely to be important as well, since endurance or high-intensity interval training was found to be more effective at increasing telomerase activity and TL than resistance training (Werner et al., 2019).

Building on previous literature we aimed to further explore the confounding intrinsic and extrinsic variables that may influence TL in athletes. Therefore, the primary objective of this study was to analyze whether a history of past concussion significantly impacted salivary TL in a large cohort of uninjured varsity athletes across a variety of sports. A secondary exploratory objective was to investigate whether patient characteristics including age, sex, type of sport (collision, contact, or non-contact), and time in season (pre-season or mid-season) influenced salivary TL. Collection of blood and cerebral spinal fluid may provide higher concentrations of the CNS biomarker of interest, but collection of these fluids can be challenging in non-clinical settings and have worse safety profiles. Saliva offers a non-invasive, readily available, inexpensive, and easily stored biofluid, containing valuable biomarkers released from the brain (Lin J. et al., 2019). Saliva has been analyzed successfully in other CNS injuries (Pietro, 2018) and therefore, was chosen as the fluid medium for this study.

## Materials and Methods

### Participants

This was a cross-sectional cohort study of athletes recruited from the University of Calgary varsity athlete population, and club sport athletes in Calgary, Alberta, Canada from January 2018–March 2019. Inclusion criteria included participation in varsity or community athletics, ages 16–27 years, and no medical history of neurological conditions such as moderate/severe traumatic brain injury, stroke, or chronic neurological conditions. The demographic information collected included age, sex, history of concussion (HOC), time in season (TIS), and sport participation. Determining sport participation was necessary to categorize the level of contact experienced by the athlete (non-contact, contact, or collision). Level of contact was determined using a classification system defined by Rice et al., and the American Medical Society for Sports Medicine (Rice, 2008; Harmon et al., 2013). In this study, *collision sport* was classified as purposeful body contact that was strategic to the sport; *contact sport* was classified as sports where bodies may come together during gameplay, but not for the sole intent of hitting; and *non-contact* were sports that do not involve contact or collision. The sports included in this study are listed in Table 1. Two separate analyses were conducted using this variable, as athletes were divided into two categories of either contact/collision or non-contact categories (CT2), and divided into three categories of contact, collision, or non-contact categories (CT3). TIS was classified as either mid-season (athletes actively playing league games in their sport) or pre-season (athletes not currently playing any league games). This study was approved by the University of Calgary Conjoint Health Research Ethics Board (REB15-1786).

**Table 1:** Participant characteristics.

	***N* = 183**	**Male**	**Female**	***P*-value**
		***N* = 108**	***N* = 75**	
Age, mean (SD)	19.7 (1.9)	19.6 (1.7)	19.9 (2.0)	0.50
Sex, n (%)				
Male	108 (59.0)			
Female	75 (41.0)			
Missing	10 (5.5)			
TL, mean (SD)	5,565.4	5,636.6	5,462.8	0.10
	(431.2)	(335.3)	(526.0)	
Number of concussions, median (Q1–Q3)	0 (0–1)	0 (0–1)	1 (0–1)	0.09
History of concussion, *n* (%)				0.06
Yes	81 (44.3)	42 (41.2)	39 (55.7)	
No	91 (49.7)	60 (58.8)	31 (44.3)	
Missing	11 (6.0)			
Collision type 2, n (%)				0.56
Contact	163 (89.1)	95 (88.0)	68 (90.7)	
Non-contact	20 (10.9)	13 (12.0)	7 (10.0)	
Collision type 3, n (%)				0.0005
Collision	129 (70.5)	85 (78.7)	44 (58.7)	
Contact	34 (18.6)	10 (9.3)	24 (32.0)	
Non-contact	20 (10.9)	13 (12.0)	7 (9.3)	
Time in season, n (%)				<0.0001
Pre-season	117 (63.9)	92 (85.2)	25 (33.3)	
Mid-season	66 (36.1)	16 (14.8)	50 (66.7)	
Sport, n (%)				
Basketball	13 (7.1)	10 (9.3)	3 (4.0)	
Field hockey	14 (7.7)	0 (0.0)	14 (18.7)	
Football	72 (39.3)	72 (66.7)	0 (0.0)	
Hockey	10 (5.5)	3 (2.8)	7 (9.3)	
Lacrosse	7 (3.8)	7 (6.5)	0 (0.0)	
Rugby	32 (17.5)	0 (0.0)	32 (42.7)	
Soccer	7 (3.8)	0 (0.0)	7 (9.3)	
Swimming	2 (1.1)	2 (1.9)	0 (0.0)	
Track and field	11 (6.0)	7 (6.5)	4 (5.3)	
Volleyball	7 (3.8)	4 (3.7)	3 (4.0)	
Wrestling	8 (4.4)	3 (2.8)	5 (6.7)	

*SD, standard deviation; n, the number of participants; Q1, first quartile; Q3, third quartile*.

### Procedure

All athletes signed informed consent prior to study participation. Saliva samples, informed consent, and past medical history were collected prior to athlete and team training sessions, or during pre-season medical health assessments with the team's sport medicine therapists. The SCAT5 was used to collect background medical history (including self-reported HOC or any psychiatric/neurological diagnoses), demographic information, and self-reported symptom evaluations using the embedded graded symptom checklist (Dessy et al., 2017).

### Saliva Collection and Analysis

Three salivettes were filled with 1 ml of saliva *via* the clean drool technique (Salimetrics, USA), between the hours of 6:00 am and 11:00 am. Athletes were asked to abstain from brushing their teeth, tobacco-use, food, and caffeine for 8 h prior to saliva collection. Collected saliva was stored at −80°C until analysis. Analysis of TL was conducted on all saliva samples using a protocol previously described (Cawthon, 2002; Hehar and Mychasiuk, 2016; Hehar et al., 2017). Genomic DNA isolated from buccal epithelial skin cells present in the saliva was extracted using the Qiagen Allprep RNA/DNA mini kit (Qiagen, Germany), according to manufacturer protocols. Concentration and purity of all samples were measured with the Nanodrop 2000 (Thermo Fisher Scientific, MA).

TL was determined using reverse-transcription polymerase chain reaction (RT-PCR). Two researchers independently performed all PCR tests and each PCR reaction was run in duplicate on a 96-well plate. Each PCR reaction contained 1 μl of gDNA in a total volume of 20 μl, using 1 × SYBR Green FastMix with Rox for qRT-PCR on a CFX Connect Real Time PCR Detection System (Bio-Rad, Hercules, CA). DNA was amplified in two separate mixtures, one with Tel primers (+) ggtttttgagggtgagggtgagggtgagggtgagggt (–) tcccgactatccctatccctatccctatccctatcccta and the other with 36B4 primers (+) cagcaagtgggaaggtgtaatcc (–) cccattctatcatcaacgggtacaa. TL was determined using the telomere to single copy ratio (T/S), where the single copy corresponds to the 36B4 gene. Based upon the computations by Cawthorn, when T/S = 1, the unknown DNA is identical to the reference DNA, with respect to telomere repeat number and single copy gene number (Cawthon, 2002). Therefore, T/S ratios greater than one exhibit an overall increase in telomere repeat number, and the opposite when T/S is less than one. The T/S ratio had been determined to be approximately equal to [2Ct(telomere) / 2Ct(36B4)] −1 = −2–ΔCt. A linear regression equation, y = 1910.5x + 4157 (where y = TL and x = −2–ΔCt) was then used to determine relative TL which was used for pre-injury TL values (Cawthon, 2002).

### Statistical Analysis

Descriptive statistics described patient characteristics and subsequent Chi square tests as well as Mann-Whitney-U tests evaluated univariate associations between categorical and continuous participants' characteristics, respectively. Multiple linear regression was used to examine the relationship between saliva TL and HOC (yes/no), number of past concussions, sport participation, and TIS (pre-season/mid-season), with additional adjustment for age and sex. Statistical analysis was performed using SAS 9.4 software. Statistical significance was considered as *p* < 0.05 and 95% confidence intervals (95% CI) were reported where applicable.

## Results and Analysis

### Participant Characteristics

**Figure 1:**
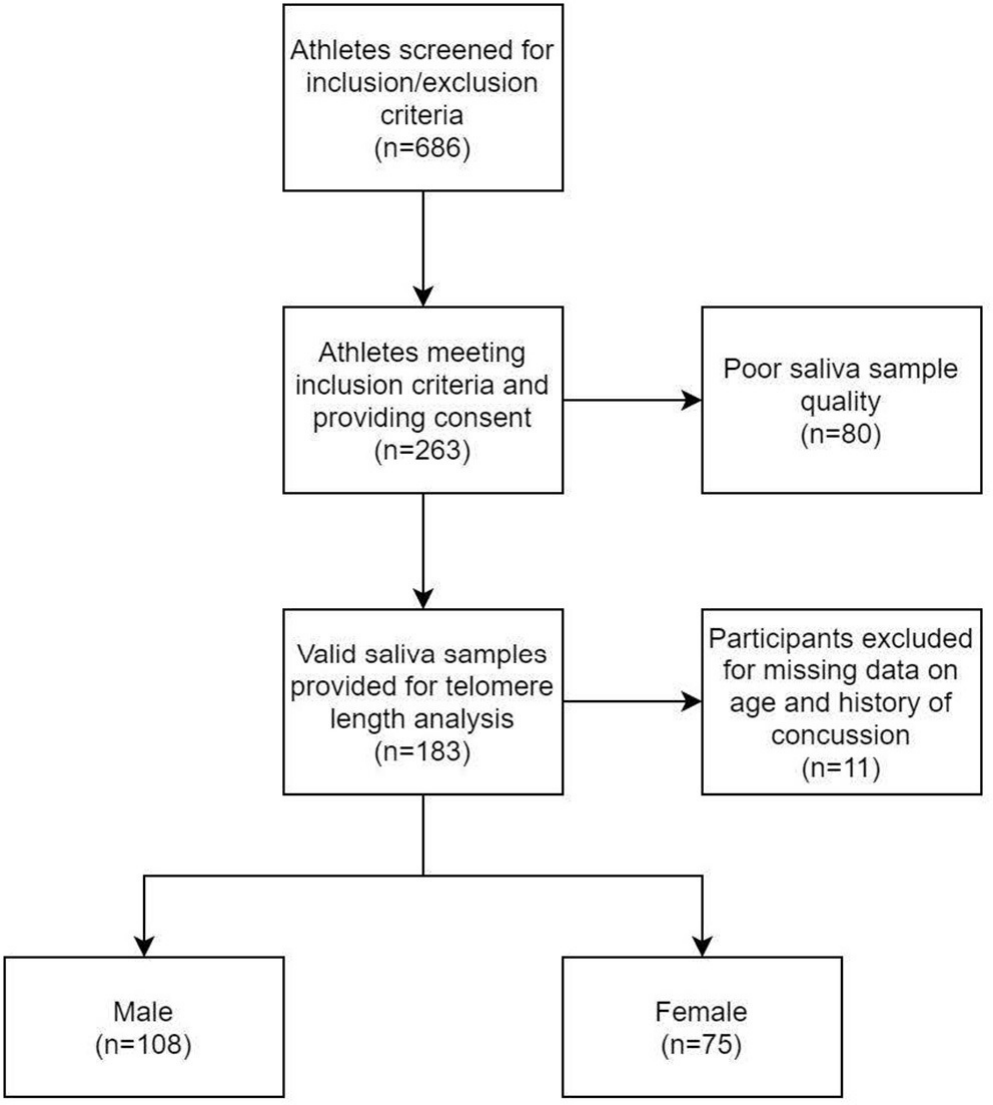
Study flowchart of participant recruitment.

**Figure 2:**
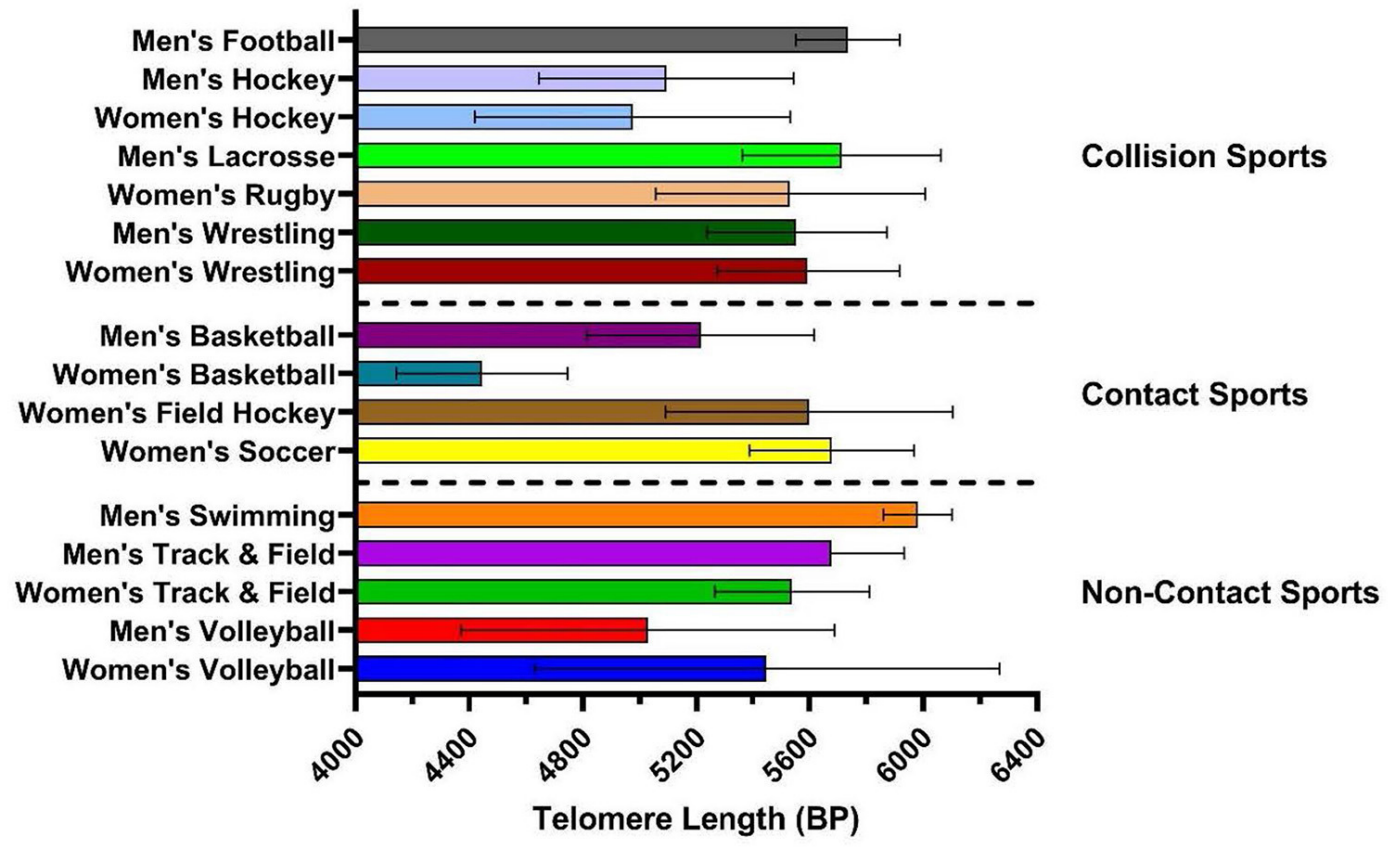
Mean telomere length (base pairs, BP) stratified by sport. Sports separated by CT3 (contact type 3) categories: (1) collision sports (purposeful body contact being strategic to the sport); (2) contact sport (bodies may come together during gameplay but not for the sole intent of hitting); and (3) non-contact sports (sports that do not involve any contact or collision). Error bars represent mean and SD of each group.

A total of 686 athletes were screened for this study of which 263 athletes met inclusion criteria and provided consent to participate. Of these, 183 provided valid saliva samples for analysis. 11 participants were excluded due to missing data on history of concussion and age (see Figure 1 for flowchart). There were 108 males (19.6 ± 1.7 years) and 75 females (19.9 ± 2.0 years), with the total sample mean age of 19.7 ±1.9 years. Overall, 44.3% of participants had a history of one or more previous concussions. Samples were taken from athletes in 11 different sports. Of these athletes, 70.5% of participants were *collision* athletes, 18.6% were *contact* athletes, and 10.9% were *non-contact* athletes respectively. Lastly, 63.9% of participants were pre-season at time of sampling, and 36.1% were in their mid-season. See Table 1 for all participant characteristics and descriptive statistics. Figure 2 shows a representation of mean TL stratified by sport and CT3 collision type, whereby mean TL in the current study's athlete population was highly variable depending on the sport played.

### Influence of History of Concussion on Telomere Length

**Figure 3:**
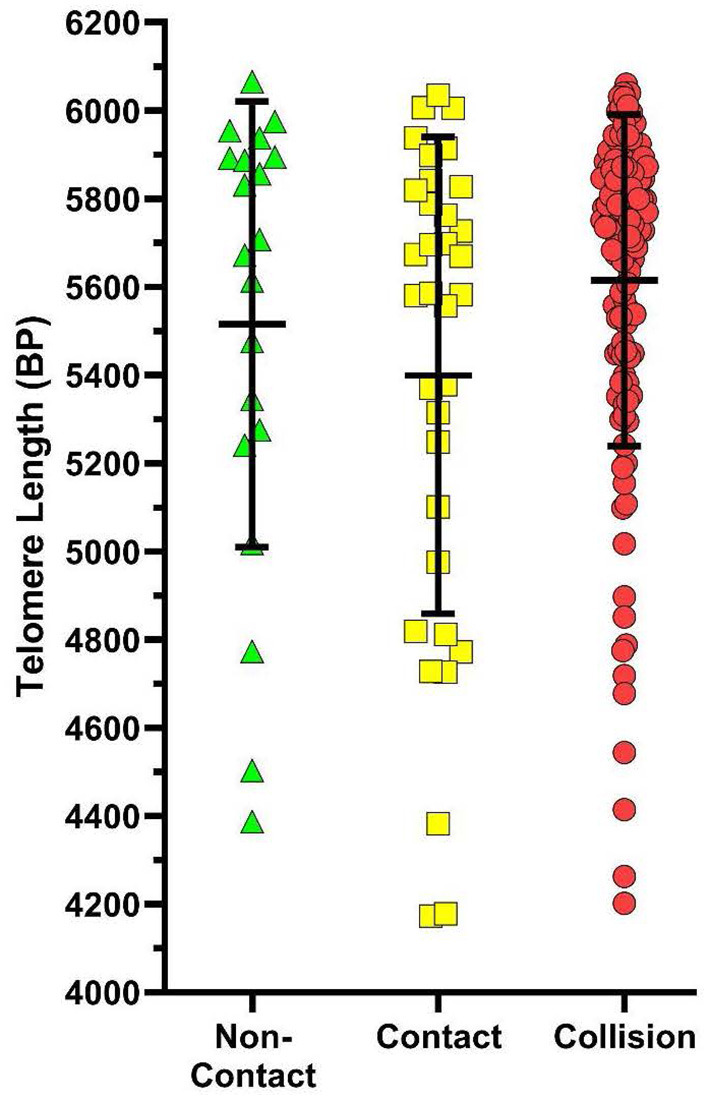
Scatterplot of telomere lengths (base pairs; BP) in non-contact sports athletes (green), contact sport athletes (yellow), and collision sport athletes (red) when sports are classified in the CT3 category. Error bars represent mean and SD of each group. While the average TL in each collision type tended to differ, no significant association was found between collision type and TL after adjusting for age, sex, TIS, and HOC [β = 199.4, 95% CI (−24.0, 422.8), *p* = 0.080].

After adjusting for age, sex, TIS, and sport participation, there was no significant relationship between TL and HOC whether grouped as CT2 [β = 4.6, 95% CI (−126.3, 135.6), *p* = 0.944] or CT3 [β = −3.5, 95% CI (−134.3, 127.3), *p* = 0.958]. An exploratory analysis was also completed investigating the effect of the number of past concussions, but found no statistical significance in CT2 [β = −10.7, 95% CI (−80.2, 58.9), *p* = 0.763] or CT3 [β = −13.6, 95% CI (−82.9, 55.7), *p* = 0.699]. Additionally, there was no significant relationship found between collision type and TL in the CT2 category [contact β = 169.8, 95% CI (−51.4, 391.1), *p* = 0.132] or in the CT3 category [contact β = 60.6, 95% CI (−198.6, 319.9), *p* = 0.645; collision β = 199.4, 95% CI (−24.0, 422.8), *p* = 0.080] after adjusting for age, sex, TIS, and HOC (see Figure 3).

**Figure 4:**
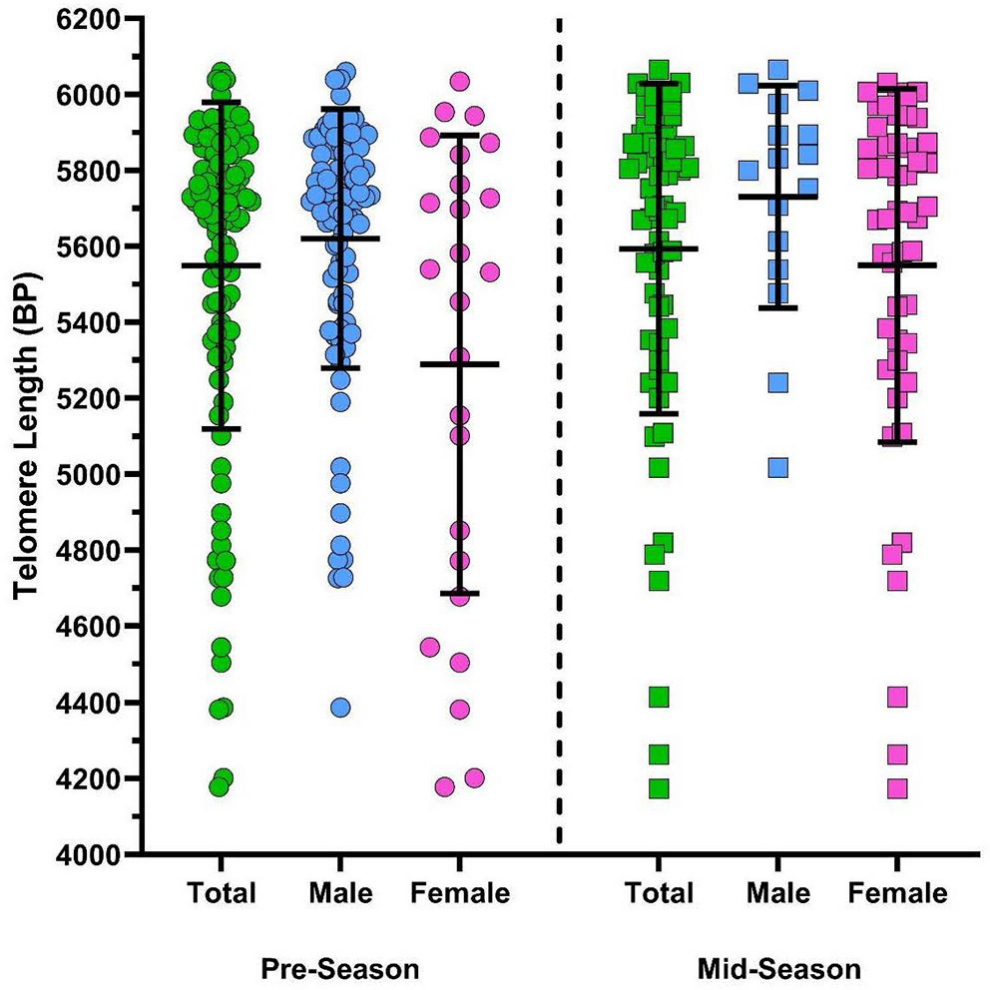
Scatterplot of athlete telomere lengths (base pairs; BP) of total (green), male (blue), and female (pink) samples collected pre-season (circles) and mid-season (squares). Error bars represent mean and SD of each group. Findings from multiple linear regression modelling indicate a significant association between TL and TIS whereby participants sampled mid-season had longer TL than participants sampled pre-season in CT3 classifications [β = 231.4, 95% CI (61.9, 401.0), *p* = 0.008] when controlling for age, sex, HOC, and collision type. A significant association was also found between sex and TL length, whereby males had longer TL compared to females in CT3 classifications [β = 284.8, 95% CI (111.5, 458.2), *p* = 0.001] adjusting for age, TIS, HOC, and collision type.

There was a significant difference in TL depending on TIS when the sample was collected (see Figure 4). The estimate of expected TL for participants who were sampled mid-season was ~252 base pairs higher compared to the expected TL for participants who were sampled in pre-season when analyzing by CT2 classification [β = 251.6, 95% CI (83.2, 420.0), *p* = 0.004] and ~231 base pairs higher in the CT3 category [β = 231.4, 95% CI (61.9, 401.0), *p* = 0.008] when controlling for age, sex, HOC, and collision type. Additionally, males were also found to have significantly larger TL than females in both CT2 [β = 330.8, 95% CI (166.6, 495.0), *p* ≤ 0.0001] and CT3 [β = 284.8, 95% CI (111.5, 458.2), *p* = 0.001] after adjusting for all other variables (see Figure 4).

## Discussion

This study explored the stability of telomere length (TL) in young healthy athletes when analyzing for multiple confounding variables. A past medical history of concussion, age, and sport contact-type did not influence TL. However, timing of sample collection (pre-season vs. mid-season) and sex significantly influenced TL. Male athletes and those athletes with saliva collected mid-season had significantly longer TL compared to females and samples collected pre-season. We concluded, when analyzing saliva TL in young athletes, sex, and time of collection, may influence results and should be considered as potential confounders.

### Relationship Between Telomere Length and History of Concussion

Previous animal research investigating TL collected from the skin following concussion has found TL to be significantly shortened post-concussion (Hehar and Mychasiuk, 2016). In contrast, when Symons et al. surveyed 97 Australian footballers, they found that although a history of concussion did not influence saliva TL, participation in contact sports did (Symons et al., 2020). The discrepancy in these studies could perhaps be due to the timing of collection in relation to concussion recency. Skin cell TL was collected in the acute/subacute period following concussion in the animal model study first discussed, and TL were significantly shorter in the concussed animals compared to shams (Hehar and Mychasiuk, 2016). In Symons et al. and the current study, all athletes with a HOC were fully recovered at the time of saliva collection (Symons et al., 2020). The lack of association between HOC and TL may be indicative of a short temporal window for saliva TL as a biomarker of SRC. Clinical studies have also found regular exercise training to upregulate telomerase reverse transcriptase expression (TERT; the catalytic subunit of the telomerase enzyme) and telomerase activity, potentially attenuating telomere attrition in those athletes post-concussion (Denham and Sellami, 2021). Our results also failed to demonstrate a dose response of previous concussions (i.e., more concussions did not influence TL), possibly due to the reasons discussed above. Our data together with previous literature suggests that concussions do not have a lasting effect on saliva TL in recovered athletes. Future studies will require sedentary control groups in addition to exercising athletes to fully explore this theory.

### The Athlete's Time in Season

When controlling for all variables, we found saliva sampled from athletes participating in sport mid-season had longer TL than saliva sampled from athletes participating in sport pre-season. Physical performance expectations are greater while actively engaging in competition, likely creating a difference in training intensity and regularity of exercise while mid-season. These findings align with previous studies showing participation in high intensity aerobic and anaerobic exercise to significantly lengthen TL (Simoes et al., 2017; Sellami et al., 2021). Athletes participating in regular high intensity aerobic exercise have upregulation of telomerase activity and telomeric repeat binding factor 2 (Arsenis et al., 2017). Exercise can also combat harmful states, by reducing reactive oxygen species (ROS) and proinflammatory cytokines which consequently reduces oxidative stress and inflammation through increased levels of anti-inflammatory cytokines and antioxidants (Arsenis et al., 2017; Sellami et al., 2021). Interestingly, ROS and free radicals are found in greater concentrations in those who are physically inactive, and TL are shortened in sedentary individuals (Magi et al., 2018). Further, elite sprinters have been found to have longer TL than age-matched inactive controls, and endurance athletes not only have longer TL but also upregulated TERT and TPP1 mRNA expression—two important genes in regulating telomere health (Denham et al., 2016; Simoes et al., 2017). The results from this study uncover the importance of considering collection time when researching fluid biomarkers in athletes.

### Sex and the Impact on Telomere Length

Contrary to previous literature, the current study found that males had a larger mean TL than females (Benetos et al., 2001; Barrett and Richardson, 2011). Longer TL in females is hypothesized to be due to various factors. One theory is that females age slower than males and have longer life expectancies (Barrett and Richardson, 2011). Given that TL serves as a “biological clock,” those who live longer are thought to have slower telomere degradation. A systematic review outlined that females produce less ROS than males, and the presence of estrogen can be neuroprotective against ROS and catalyze greater telomerase activity (Gardner et al., 2014). Together, these are just two potential mechanisms explaining the trend of longer TL in females compared to males. Our conflicting results could be due to the type of tissue analyzed, the age of the participants sampled, or not accounting for hormonal changes (Quinn et al., 2021). Animal model research has found males to have longer TL in all organ tissue except for the brain (Cherif et al., 2003). Salivary epithelial buccal cell DNA is thought to derive from ectoderm, sharing a common lineage with brain tissue. Therefore, it is possible that TL was not longer in our female athletes due to the source of DNA analyzed in this study. Secondly, although women are documented to have longer TL on average, a systematic review by Gardner et al. identified that sex-based population differences in TL do not appear until ~30 years of age, which is outside of the age range included in this study (Gardner et al., 2014). Lastly, TL has been found to be significantly linked to hormonal levels in females, specifically estrogen and progesterone (Lee et al., 2005; Wunderle et al., 2014; Shin et al., 2016). We did not record time of menses in the athletic population sampled (Quinn et al., 2021), a limitation of this study. Further, we did not ask about regularity of menses or signs and symptoms of Relative Energy Deficiency in Sport (RED-S), a syndrome that can affect female athletes training at high-intensity due to an imbalance of dietary energy intake and energy expenditure (previously known as the Female Athlete Triad, but has now been expanded to include multiple aspects of physiological function in athletes) (Nattiv et al., 2007; Mountjoy et al., 2014). Therefore, it is unclear if the difference in TL due to sex is influenced by altered menstrual cycles in the female athletes. Future studies exploring TL in athletes should collect data on time in menstrual cycle, regularity of menses, intensity of training and a diagnosis of the RED-S.

### Sport Contact-Type and Telomere Length

This study found sport contact-type did not influence TL in healthy athletes. Analysis of contact-type was based on criteria determined using a classification system defined by Rice et al., and the American Medical Society for Sports Medicine (Rice, 2008; Harmon et al., 2013). Sports were classified as (1) *collision*, purposeful body contact that was strategic to the sport; (2) *contact*, where bodies may come together during gameplay, but not for the sole intent of hitting; and (3) *non-contact*, sports that do not involve contact or collision. Our results conflict with findings by Symons et al. that showed saliva TL to be significantly shorter in Australian Rules Football players, considered a contact sport, compared to athletes playing non-contact sports (Symons et al., 2020). The discrepancy between the two studies could be due to multiple reasons. First, high intensity aerobic and anaerobic activity can influence TL. Previous studies have described high intensity cardiovascular sports, particularly long distance running, is associated with longer TL (Lin X. et al., 2019). Furthermore, high intensity power sports using greater muscle contraction, were found to have greater immune-inflammatory and oxidative stress markers, and less anti-inflammatory profiles than lower power sports, potentially influencing TL (Mitchell et al., 2005; Sohail et al., 2020). Both studies divided athletes into contact versus non-contact but did not take into consideration the aerobic or anaerobic intensity required for the sport participation. It is unclear if Australian Rules Football requires a different level of aerobic exercise training than the contact sports collected for this study. Furthermore, the absolute volume and ratio of aerobic/anaerobic training in different sports may need consideration when investigating TL. Lastly, the percentage of time a collision-sport player may be exposed to full-contact gameplay may vary between sports and sport level given different body-contact policies in practices and games, likely contributing to differences seen across sports (Black et al., 2017). Future studies assessing TL should collect data on aerobic and anaerobic activity as both have been shown to influence TL, in addition to the time spent in contact situations (i.e., competitive games versus non-contact practice).

### Age and Telomere Length

TL is a marker of cellular age and is well documented to have an inverse relationship with increasing age (Benetos et al., 2001). Interestingly, this study did not find that age significantly influenced TL. This is most likely due to the narrow age range of athletes included in this study which consisted of varsity and club athletes aged 16–27. Telomeres are thought to shorten ~30–200 base pairs per year, so age becomes more significant the older an individual becomes (Starkweather et al., 2014). Additionally, on average, the brain does not finish developing until one's mid-to-late twenties (Johnson et al., 2009). Therefore, salivary TL changes may not be reflected until later in life, after full maturation of the brain has occurred. This notion is supported by findings from a study investigating TL differences by sex. It is well established in the literature that adult females have, on average, longer telomeres than males (Gardner et al., 2014). However, this relationship is not evident until after ~30 years of age, beyond the age-range of this study's participants. Future studies may benefit from investigating the impact of HOC on TL in varsity and club athletes 10 or more years after their sport participation, as differences may appear when their brain has finished developing. TL damage may also occur due to increased oxidative stress and inflammation throughout normal aging after neurodevelopment is considered complete (Eitan et al., 2014; Franceschi and Campisi, 2014). Findings from a recently published study investigating age-dependent changes in pro-inflammatory markers and TL suggest that athletes older than 25 do not benefit from the protective effects of exercise on systemic inflammation and telomere health when compared to younger cohorts (Sellami et al., 2021). If older professional or master athletes were included in the present study, it is expected that age may have negatively influenced TL.

### Justification of Saliva as a Source for CNS Biomarkers

Although fluid biomarkers are obtained in the highest concentrations in cerebral spinal fluid or blood, these methods are invasive, potentially difficult to collect, and may add increased safety risk to participants. Saliva offers a non-invasive, readily available, inexpensive, and easily stored biofluid that can contain biomarkers of CNS injury (Lin J. et al., 2019). Following a CNS injury like a SRC, the blood brain barrier, normally highly selectively permeable, becomes compromised (Aaron Dadas, 2018). This allows molecules normally only present in the CNS to diffuse outwards and influence peripheral tissues and fluids (Aaron Dadas, 2018). In this way, it is believed that neuronal damage can influence peripheral skin cells of the mouth, and other peripheral tissues (Hehar and Mychasiuk, 2016). Therefore, TL sampled from peripheral tissue would be influenced by CNS injury. Although the numerical value of TL will vary depending on the tissue it is sampled from, past research has shown linear correlations between the TL in different tissues (Demanelis et al., 2020). In a study of elderly patients, TL sampled from blood leukocytes, synovial tissue, and skin cells illustrated linear correlation, indicating a potential holistic mechanism of control on telomere maintenance throughout the body, and the ability of multiple tissue types to represent relative TL of the individual (Friedrich et al., 2000). Similar results were found in the blood, buccal cells, and fibroblasts of a cohort of individuals with inherited bone marrow failure syndromes, where strong intra-individual correlations were observed, and damage was reflected across multiple body tissues (Gadalla et al., 2010). Therefore, saliva though “downstream” from the CNS, has the capacity to reflect CNS injury, specifically through changes in TL.

### Limitations

There are important limitations to consider when interpreting this data. First, the results are likely impacted by the unequal balance of athletes participating in contact/collision sports compared to non-contact sports. Our study was likely underpowered to detect statistically significant associations in TL and contact sport classification after adjusting for all other variables. With a larger non-contact sample size, it is possible that greater differences would have been observed, and the impact of participating in contact/collision sports on TL may have been consistent with other literature. Future studies should also collect information on aerobic and anaerobic training volumes across sports to be able to stratify TL analyses by physical activity levels between sports using validated measures [e.g., the international physical activity questionnaire (IPAQ)] (Craig et al., 2003). We also did not explore the relationship between TL and psychosocial factors such as mental health, sleep, or stress. Robust findings have demonstrated a strong association between psychological stress and depression and shorter TL (Liu et al., 2017). In females, TL shortening was associated with the severity of depression, while in males it was negatively associated with amount of peer social interaction and tendency to use worrying as a coping mechanism. As over 30% of university students experience some form of depressive disorder, future studies should likely take psychological health into account (Liu et al., 2017; Dinis and Bragança, 2018). A clear relationship also exists between poor sleep quality and shorter TL. In a cohort of post-menopausal women, having seven hours or more of sleep each night was found to be associated with longer TL compared to women who slept less than seven hours (Grieshober et al., 2019). Additionally, subjectively restful sleeps, and feeling as if one is well rested, have also been associated with longer TL (Iloabuchi et al., 2020). Given that up to 60% of university/college students may experience inadequate quality of sleep, this could substantially influence TL results (Schlarb et al., 2017). Lastly, we did not collect female hormone or menstrual cycle data to interrogate the possible influence of RED-S on TL of female athletes in this population (Mountjoy et al., 2014).

## Conclusion

Saliva offers a non-invasive, readily available source of fluid biomarkers that may reflect CNS injury. This study examined the associations between saliva TL and multiple confounding variables in healthy varsity athletes to inform on its potential use in SRC management. While results showed no significant associations between TL and history of concussion, age, or sport contact-type, we found that timing of sample collection and sex influenced saliva TL in athletes across multiple sports. Athletes sampled mid-season displayed longer TL on average compared to those sampled pre-season, perhaps reflecting the physiological effects of different training contexts or regimes. Females also had shorter TL than males, potentially due to hormonal differences or the presence of the Female Athlete Triad/RED-S which was not accounted for in this study. Female athletes in sports where an emphasis is place on weight and/or leanness for performance or competition categories (e.g., wrestling or track and field for this study) are thought to have increased vulnerability to the Triad, possibly influencing TL data (Mountjoy et al., 2014). Overall, our findings suggest that multiple intrinsic and extrinsic variables must be considered when evaluating the utility of saliva TL as a fluid biomarker for SRC given their influence on data sampled from a healthy athlete population.

## Data Availability Statement

The raw data supporting the conclusions of this article will be made available by the authors, without undue reservation.

## Ethics Statement

The studies involving human participants were reviewed and approved by University of Calgary, Conjoint Board of Ethics Approval. The patients/participants provided their written informed consent to participate in this study.

## Author Contributions

All authors listed have made a substantial, direct, and intellectual contribution to the work and approved it for publication.

## Funding

This study was funded by the Hotchkiss Brain Institute, Calgary, Alberta, Canada and supported by the Canadian Institutes of Health Research (CIHR) Grant Number: PJT-153051. Author JT was also funded by CIHR.

## Conflict of Interest

The authors declare that the research was conducted in the absence of any commercial or financial relationships that could be construed as a potential conflict of interest.

## Publisher's Note

All claims expressed in this article are solely those of the authors and do not necessarily represent those of their affiliated organizations, or those of the publisher, the editors and the reviewers. Any product that may be evaluated in this article, or claim that may be made by its manufacturer, is not guaranteed or endorsed by the publisher.
